# Anthropometric, Cardiopulmonary and Metabolic Benefits of the High-Intensity Interval Training Versus Moderate, Low-Intensity or Control for Type 2 Diabetes: Systematic Review and Meta-Analysis

**DOI:** 10.3390/ijerph16224524

**Published:** 2019-11-15

**Authors:** Ivan Lora-Pozo, David Lucena-Anton, Alejandro Salazar, Alejandro Galán-Mercant, Jose A. Moral-Munoz

**Affiliations:** 1 Department of Nursing and Physiotherapy, University of Cadiz, 11009 Cadiz, Spain; ivan.lorapozo95@gmail.com (I.L.-P.); alejandro.galan@uca.es (A.G.-M.); joseantonio.moral@uca.es (J.A.M.-M.); 2 Department of Statistics and Operational Research, University of Cadiz, 11009 Cadiz, Spain; alejandro.salazar@uca.es; 3 Institute of Research and Innovation in Biomedical Sciences of the Province of Cadiz (INiBICA), University of Cadiz, 11009 Cadiz, Spain; 4 Observatory of Pain, Grünenthal Foundation-University of Cadiz, 11009 Cadiz, Spain

**Keywords:** high-intensity interval training, physical activity, physical exercise, type 2 diabetes

## Abstract

This study aims to evaluate the effectiveness of high-intensity interval training compared with no intervention and other types of training interventions for people with Type 2 diabetes. A systematic review and meta-analysis of randomized controlled trials that used high-interval intensity training to improve anthropometric, cardiopulmonary and metabolic conditions were conducted. The search was performed during October–December 2017 using the databases PubMed, Web of Science and Physiotherapy Evidence Database (PEDro). The methodological quality of the studies was evaluated using the PEDro scale. A total of 10 articles were included in this meta-analysis. After statistical analysis, favorable results were obtained for high-Intensity Interval Training compared with control (non-intervention): [Weight: Standardized mean difference (SMD) = −2.09; confidence interval (CI) 95%: (−3.41; −0.78); body-mass index: SMD = −3.73; CI 95%: (−5.53; −1.93); systolic blood pressure: SMD = −4.55; CI 95%: (−8.44; −0.65); VO_2max_: SMD = 12.20; CI 95%: (0.26; 24.14); HbA_1c_: SMD = −3.72; CI 95%: (−7.34; −0.10)], moderate intensity continuous training: [body-mass index: SMD = −0.41; CI 95%: (−0.80; −0.03); VO_2max_: SMD = 1.91; CI 95%: (0.18; 3.64)], and low intensity training: [Weight: SMD = −2.06; CI 95%: (−2.80; −1.31); body-mass index: SMD = −3.04; CI 95%: (−5.16; −0.92); systolic blood pressure: SMD = −2.17; CI 95%: (−3.93; −0.41); HbA_1c_: SMD = −1.58; CI 95%: (−1.84; −1.33)]. The results show that high-intensity interval training can be a useful strategy in order to improve anthropometric, cardiopulmonary and metabolic parameters in people with Type 2 diabetes. Despite this, it could be essential to clarify and unify criteria in the intervention protocols, being necessary new lines of research.

## 1. Introduction

Type 2 diabetes (T2D) is the most common form of diabetes in adults, and it is becoming more frequent among children and adolescents [1]. According to the International Diabetes Federation, in 2017, the diabetic population was around 425 million people worldwide and it will increase by 48% in 2045. Furthermore, 90% of the current diabetic population have T2D [2]. The etiology of T2D is multi-faceted nonetheless, there are modifiable factors such as overweight, obesity, a sedentary lifestyle, overweight [3], physical inactivity [4], smoking and alcohol consumption [5]. It should be noted that a sustained weight loss (more than 3%) can lead to clinically significant benefits thanks to the lowering of the triglyceride, the blood sugar and hemoglobin A1c (HbA_1c_) levels [6].

Regarding the pharmacological treatment of T2D, medications usually aim to lower the high levels of blood sugar, although new multifactorial approaches are emerging. These are not glucocentric and can be of great use for the prevention of diabetes complications [7]. As for physical exercise, it has been shown that it improves insulin sensitivity and lowers the blood sugar level within a desirable range [4]. It is recommended to practice 150 min of physical exercise at moderate intensity [40–60% of maximum oxygen uptake (VO2max)] or 75 min at a higher intensity (60–85% VO2max) per week in order to maintain or improve the health condition [8]. These workouts consist of long duration cardiovascular exercise at a moderate intensity without breaks, the so-called moderate intensity continuous training (MIT) [9]. Another type of training is low intensity training (LIT), which uses less intensity than MICT [8]. Currently, high-intensity interval training (HIIT), which lies in performing short intervals of exercise at a high intensity and intervals at a lower intensity or even breaks, aims to increase the fat loss in a shorter time of execution. It is of great use since time is often one of the reasons why people do not practice sport [9,10]. Recently, several meta-analyses on the effects of the practice of HIIT have been published. Ballesta García et al. [11] show that the practice of HIIT causes improvements in VO2max in subjects with coronary artery disease and heart failure, while Liu et al. [12] show that HIIT causes more improvements than MIT in the cardiorespiratory parameters in subjects suffering T2D.

Despite this, there is currently little evidence on the frequency, intensity, time and type of exercise most recommended for T2D [13], and further research in this field is needed. The general objective of this study is to evaluate the effectiveness of HIIT in patients with T2D. The specific objective is to determine whether HIIT causes a significant improvement of the anthropometric, cardiopulmonary and metabolic values compared to control (CON) or other types of physical exercise, such as MIT and LIT.

## 2. Materials and Methods

This systematic review and meta-analysis were planned and conducted according to the PRISMA statement (Preferred Reporting Items for Systematic Reviews and Meta-Analyses) for randomized clinical trials (RCTs) [14]. The protocol is registered in the PROSPERO database (CRD42018102313).

### 2.1. Search Strategy

The search of the scientific literature was carried out between October and December 2017, including the following databases: PubMed, Physiotherapy Evidence Database (PEDro) and Web of Science (WoS) (Table S1).

### 2.2. Selection Criteria

We included the RCTs published in English and Spanish from 2000 to 2017. To establish the inclusion criteria, we used the PICO model [15]: (Population): T2D with or without co-morbidity, such as hypertension, cardiovascular diseases, obesity, renal diseases, among other chronic related conditions; (Intervention): HIIT intervention. Because there is not as yet a clear consensus regarding the exercise modalities and dose of the different variables involved to prescribe HIIT (e.g., intensity and duration of the interval works and rest periods, between-series recovery duration, number of series and repetitions) [16], we have considered the definition and intensity proposed by García-Hermoso et al. [16] in their meta-analysis. In this way, HIIT is defined as a training performing intervals of exercise at a high intensity mixed with brief intervals at a lower intensity or even breaks. Concerning the intensity, we have considered the same vigorous intensity used in their review (64–90% VO2max or 77–95% heart rate max). In addition, we included HIIT with an intervention period of at least 2 weeks; (Comparison): the training programs were divided into 3 groups according to their intensity, in line with García-Hermoso et al. [16]: HIIT, MIT and LIT, so that the comparative interventions were CON, MIT, and LIT The outcomes included anthropometric variables such as body weight, body mass index (BMI) and percentage of body fat, as well as cardiopulmonary variables, such as systolic blood pressure (BP), diastolic BP, VO2max and heart rate (HR), and metabolic variables, such as HbA_1c_.

The exclusion criteria were studies in which the sample included patients with T2D and other diseases, and the outcomes were not detailed separately for each population.

### 2.3. Selection Process and Data Extraction

Firstly, we carried out a search combining the keywords in different databases. Then, we identified the potentially relevant articles by reading their titles and abstracts, removing duplicated papers. Subsequently, we carried out a thorough verification of compliance with the inclusion criteria.

Two reviewers (I.L.P. and J.A.M.M.) participated actively and independently in the selection process, the review and the systematic extraction of the data of each study included in this review. An additional reviewer (A.L.) took part in the resolution of discrepancies. The following information was extracted from each article: author; publication date; characteristics of the participants (number of subjects in the groups and their sex, average age, disease evolution, average weight and height and presence of comorbidity); and characteristics of the interventions (session type, frequency, total program duration, intervention duration, outcome and measuring instrument).

### 2.4. Assessment of the Methodological Quality of the Studies

For the assessment of the methodological quality of the studies included in this review, we used the PEDro scale [17], which is based on the Delphi list developed by Verhagen et al. [18]. A study with a PEDro score equal to or higher than 6 was considered to have a high-quality level (6–8: good; 9–10: excellent), and a study with a score of 5 or less was considered to have a low-quality level (4–5: acceptable; <4: poor) [19].

### 2.5. Statistical Analysis

A meta-analysis was carried out to compare changes in the effect size (pre- and post-intervention) between the intervention group (HIIT) and the comparison group (CON, MIT or LIT). The studies were grouped according to the outcome measure, the intervention and the comparison group. For each meta-analysis, the standardized mean difference was calculated, along with the 95% confidence interval (CI). The significance level was set at *p* < 0.05. Heterogeneity was determined by the chi-square test and the I2 statistic. When homogeneity was observed, a fixed-effect model was used. In the case of heterogeneity, a random-effects model was used. All statistical analyses were carried out by using the statistical software Review Manager (RevMan) 5.3 (The Cochrane Collaboration, The Nordic Cochrane Centre, London, United Kingdom). The results are presented in Forest plots.

## 3. Results

**Figure 1:**
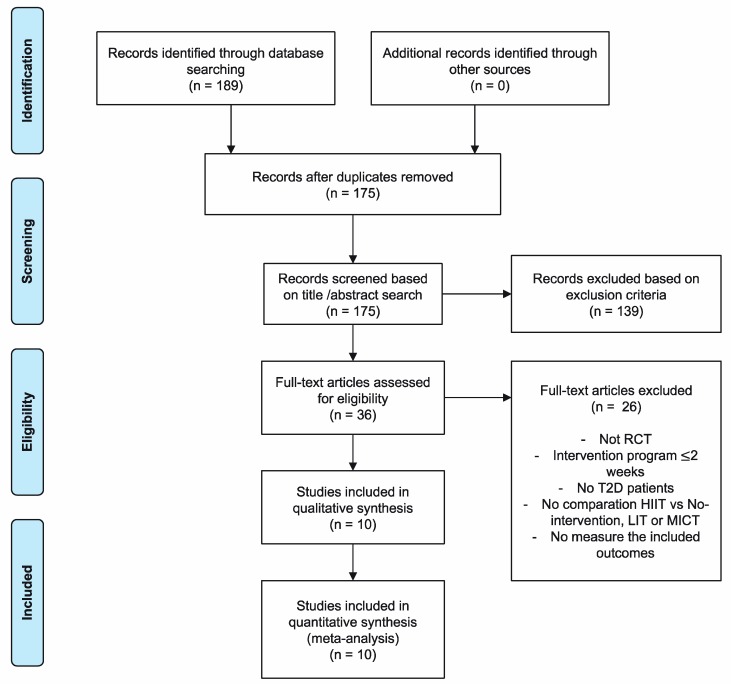
Flowchart.

As shown in the flowchart (Figure 1), after searching in different databases, we obtained a total of 189 potential articles and, after verifying the exhaustive compliance with the inclusion criteria, 10 RCTs were included in the review and subsequent meta-analysis.

### 3.1. Assessment of the Methodological Quality of the Studies

Table 1 shows the results obtained after applying the PEDro scale to the clinical trials. We consider that three [20,21,22] of the selected studies had a “good” methodological quality, as their scores were between 6–8. The remaining seven [23,24,25,26,27,28,29] studies obtained a score of 5, the lowest score, so they had an “acceptable” methodological quality. This may be due to the difficulty of conducting double-blind studies. The highest score obtained was 7 [22].

### 3.2. Characteristics of the Studies

All the studies included adult patients, with a minimum average age of 43 years [21]; 54.3% of them were male and 45.7% female. Table 2 shows the main characteristics of the participating subjects.

As for the intervention characteristics, all the studies used the HIIT intervention compared to CON: 4 RCTs [20,21,23,24], MIT: 5 RCTs [22,25,26,27,28]; LIT: 3 RCTs [20,23,29]. Table 3 sets out the main features of the interventions conducted in the different studies.

### 3.3. Groups and Subgroups Included in the Meta-Analysis

**Figure 2:**
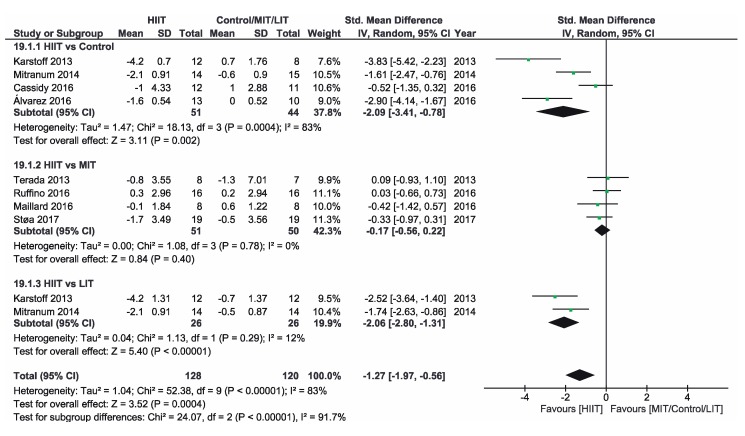
Forest plot for body weight. HIIT—high-intensity interval training, MIT—moderate-intensity training, LIT—low-intensity training, CI—confidence interval, IV—inverse variance, SD—standard deviation.

**Figure 3:**
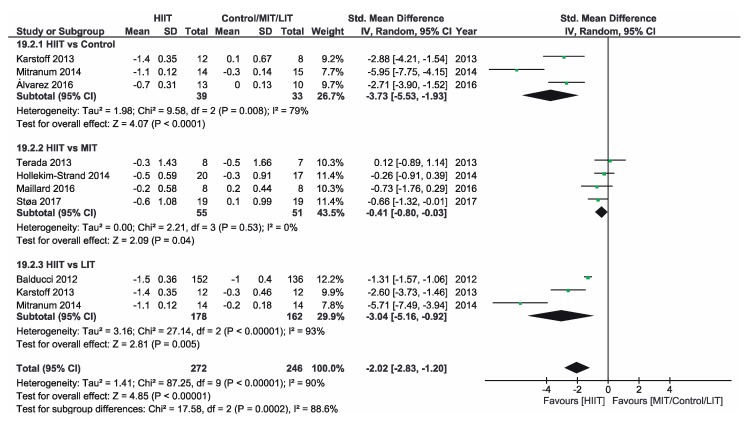
Forest plot for BMI. HIIT—high-intensity interval training, MIT—moderate-intensity training, LIT—low-intensity training, CI—confidence interval, IV—inverse variance, SD—standard deviation.

**Figure 4:**
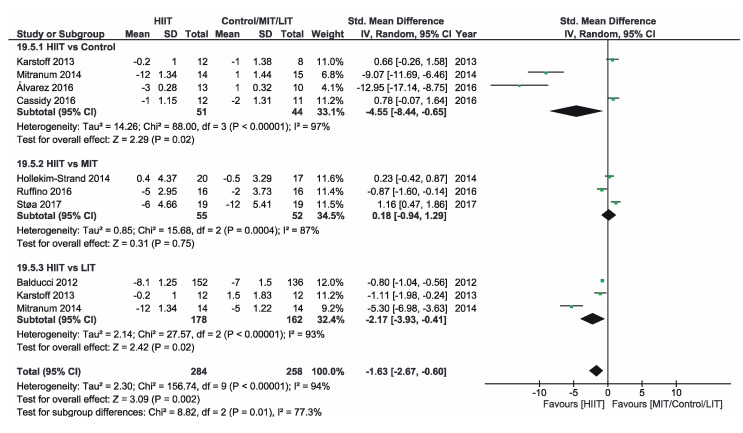
Forest plot for systolic BP. HIIT—high-intensity interval training, MIT—moderate-intensity training, LIT—low-intensity training, CI—confidence interval, IV—inverse variance, SD—standard deviation.

**Figure 5:**
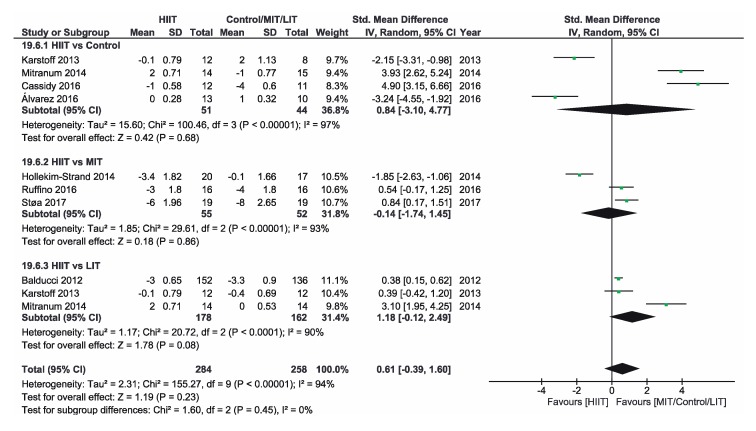
Forest plot for diastolic BP. HIIT—high-intensity interval training, MIT—moderate-intensity training, LIT—low-intensity training, CI—confidence interval, IV—inverse variance, SD—standard deviation.

**Figure 6:**
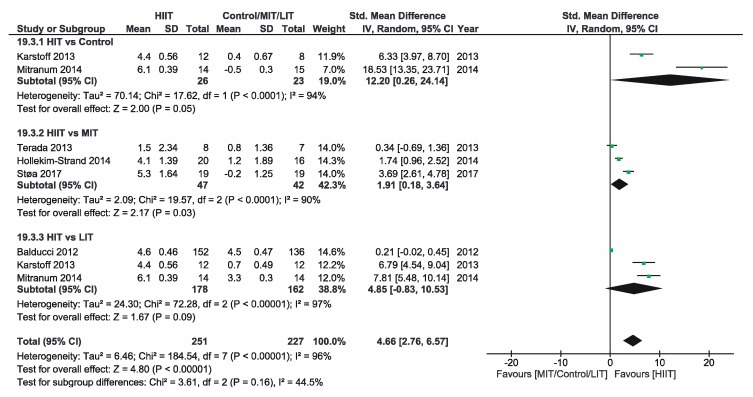
Forest plot for VO2max. HIIT—high-intensity interval training, MIT—moderate-intensity training, LIT—low-intensity training, CI—confidence interval, IV—inverse variance, SD—standard deviation.

**Figure 7:**
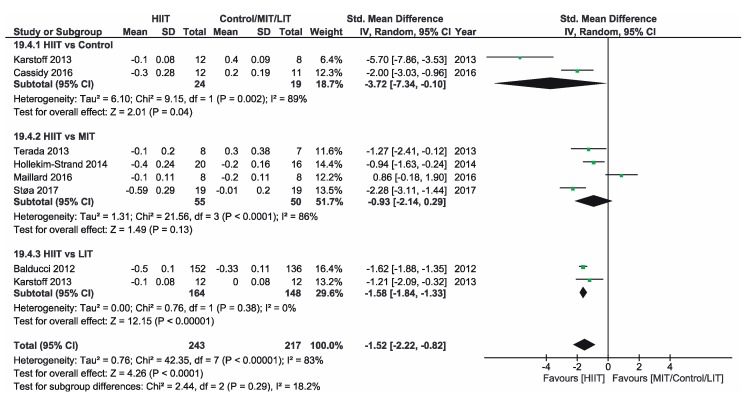
Forest plot for HbA_1c_. HIIT—high-intensity interval training, MIT—moderate-intensity training, LIT—low-intensity training, CI—confidence interval, IV—inverse variance, SD—standard deviation.

Table S2 shows the three groups for the meta-analysis according to the type of interventions, comparison groups and outcomes. Finally, Figure 2, Figure 3, Figure 4, Figure 5, Figure 6 and Figure 7 show the results obtained after the meta-analysis.

### 3.4. HIIT vs. CON

Firstly, analyzing the effects on body weight, the results showed that HIIT caused significant improvements compared to the CON group, which did not receive intervention. The study of Karstoff et al. [20] was the one that had a major effect on weight. The same applies for the improvement of BMI. The HIIT intervention proved to be more effective than CON group. In this sense, the study conducted by Mitranum et al. [23] had the greatest effects. As for the effects on systolic BP, we observed that HIIT turned out to be more effective than the CON group, as the studies that achieved the most significant effects were those conducted by Mitranum et al. [23] and Álvarez et al. [30]. This is not valid for the diastolic BP, as here the meta-analysis did not provide conclusive data. The HIIT group turned out to be more effective than the CON group regarding the effects on VO2max and the percentage of HbA_1c_. The studies of Mitranum et al. [23] and Karstoff et al. [20] are the ones that caused the most significant effects on both variables.

### 3.5. HIIT vs. MIT

As for the effects on body weight, we can observe that the studies of Terada et al. [22] and Ruffino et al. [25] showed that MIT intervention had a more favorable effect than HIIT intervention, while the studies of Maillard et al. [27] and Støa et al. [26], showed that HIIT intervention was the most favorable. However, none of the interventions produced a significant improvement. The overall result did not provide conclusive data. Regarding BMI, HIIT intervention turned out to be more effective than MIT, as shown by the studies of Hollekim-Strand et al. [28], Maillard et al. [27] and Støa et al. [26]. Terada et al. [22] showed that MIT was more effective than HIIT. As for the benefits on systolic and diastolic BP, there were no favorable results of HIIT intervention compared to MIT, and the results of both meta-analyses were not conclusive, As for the benefits on systolic and diastolic BP, there were no favorable results of HIIT intervention compared to MIT, and the results of both meta-analyzes were not conclusive. As for the effects on VO2max, the three studies showed an improvement of this parameter in the HIIT group, and the results of Hollekim-Strand et al. [28] and Støa et al. [26] were significant. The overall outcome of the meta-analysis was favorable, and it showed that HIIT intervention was more effective than MIT for the improvement of VO2max. Finally, regarding the percentage of HbA_1c_, the result of the meta-analysis was not conclusive.

### 3.6. HIIT vs. LIT

Regarding the effects on body weight, HIIT intervention turned out to be more effective than LIT and, as we can see in the study of Karstoff et al. [20], HIIT caused a significant improvement. Regarding BMI, HIIT is once again more effective than LIT, as the outcome of the meta-analysis is favorable, and the study of Mitranum et al. [23] was the one with the most significant benefits. For systolic BP, the overall result of the meta-analysis was favorable. On the other hand, the results were inconclusive for diastolic BP. As for the effects on VO2max, the overall result of the meta-analysis did not provide conclusive data. Finally, regarding the percentage of HbA_1c_, we obtained favorable results on HIIT effectiveness compared to LIT. The study of Balducci et al. [29] was the one that has the most significant effect on this variable.

### 3.7. Overalls

Concerning the effects of HIIT interventions compared to all other interventions, the overall result of the meta-analysis performed shows favorable results for body weight, BMI, systolic BP, VO2max and HbA_1c_. On the other hand, the results were inconclusive for diastolic BP.

## 4. Discussion

The purpose of this study was to synthesize through meta-analysis the scientific evidence of HIIT therapy for people suffering T2D compared to CON and other types of training, such as MIT and LIT. To do this, we used a rigorous methodology that allows the inclusion of RCTs that analyse the parameters of interest consistently, despite the heterogeneity in the implementation and the design of the different exercise programs.

There is an undeniable connection between T2D and obesity, since the risk of developing T2D increases with the degree of obesity [31]. Moreover, obesity is not only a problem in adulthood, but also in adolescence, so that interventions focused on promoting healthy lifestyles, including physical exercise, are currently of interest for public health [32]. These interventions may be performed not only in hospitals, but also at home, as they could improve the patients’ adhesion to this type of treatment [33]. The outcomes of this review suggest that HIIT intervention, in comparison to MIT, LIT and CON, turns out to be effective in the improvement of the anthropometric conditions (body weight, BMI), in the cardiovascular conditions (VO2max) and the metabolic conditions (HbA_1c_) in subjects with T2D. These results correspond partially with the results obtained by Liu et al. [12] regarding the improvements on VO2max, although they differ in the fact that that meta-analysis did not find improvements in the body weight, BMI and HbA_1c_.

In the bodyweight analysis, we observed that HIIT intervention turned out to be more effective compared to CON and LIT, even if it did not prove to be more effective than MIT. The study by Støa et al. [26] showed favorable improvements for HIIT compared to MIT. They conducted a follow-up of 3 sessions per week for 12 weeks and each session was about 52 min, unlike the other three articles of the group, whose average session duration was between 10 and 30 min. On the other hand, the study by Karstoff et al. [20], stands out for its most significant effect. They conducted 5 sessions per week, instead of 3, and similar to the rest of the studies of both groups, 16 weeks of follow-up and a greater session length (60 min). It should be noted that the studies differ in the frequency of HIIT application and follow-up time, but they have approximately the same application time. Because of this, we think that the application time could be a determining factor, but further research is needed to prove this.

Regarding BMI, it should be noted that rehabilitation through HIIT turned out to be more effective than the other three interventions, and the study of Balducci et al. [29] is the one with the most significant effects. This study stands out for having a large sample (*n* = 136) and a long effect observation time (12 months). This is why its effects should be especially taken into account. In addition, this study combines HIIT with strength training, so we can think that this combination can be favorable for BMI reduction in people with T2D.

The prevalence of Arterial Hypertension (>140/90 mmHg) in T2D ranges between 40% and 60%. Its treatment is essential to prevent cardiovascular diseases and to slow down the progression of kidney disease and retinopathy [34]. A reduction of 2.1/0.9 mmHg in BP may reduce the occurrence of cardiovascular diseases by up to 10% [30]. In our study, regarding systolic BP, we noted that HIIT turned out to be more effective than CON and LIT. HIIT [23,25,30] and MIT [26,28] interventions are the most effective in reducing this variable. As for diastolic BP, HIIT did not turn out to be more effective than the other interventions. Regarding the results of HIIT intervention compared to MIT, it can be hypothesized that the comorbidity presented by the patients may have affected the results of this outcome, making it different from the other studies without comorbidity. Our results do not prove that this is the underlying reason. On the one hand, it is known that exercise training can improve diastolic BP in patients with left ventricular diastolic dysfunction [35]. Given that left ventricular dysfunction is the comorbidity considered in the study by Hollekim-Strand et al. [28], it is reasonable to think that this might be the cause of its impact on this particular subgroup. On the other hand, we also think that the intervention conducted by these authors could be an effective strategy when we have diastolic comorbidity associated with T2D.

Cardiovascular exercises are essential to keep optimal cardiovascular health. VO2max indicates the maximum capacity of the cells of our body to absorb and use oxygen [36], and it is also a good predictor of the glucose disposal by plasma insulin [23]. The outcome of the three groups showed that HIIT is an effective strategy to increase the absorption of VO2max. It is worth mentioning the studies by Støa et al. [26] and Hollekim-Strand et al. [28], which follow a similar HIIT strategy based on intervals of 4 × 4 min at an intensity of 90–95% of maximum HR, an intervention duration of 12 weeks and significant results of HIIT compared to MIT. Furthermore, Mitranum et al. [23] show significant results in comparison to CON and LIT. The results obtained in our study suggest that HIIT is more effective than MIT on the improvement of VO2max, but we cannot conclude that HIIT is more effective than LIT. However, the results suggest so, although statistical significance was not obtained. A possible reason for the lack of significance is that the study of Balducci et al. [29], the one with larger sample size, did not report significant differences between HIIT and LIT. Nevertheless, the limitations of their study may have influenced the results, since the authors suggest that both intervention groups achieved only 1/3 of the physical activity at different intensities, and 15–20% differences in intensity between groups could not produce clinically relevant differences. On another note, in the studies of Støa et al. [26] and Mitranum et al. [23], the basal cardiovascular fitness of the patients was very low, so it might suggest that HIIT is an effective strategy when patients do not have optimal levels of cardiorespiratory fitness.

Regarding HbA_1c_, patients with T2D tend to have higher levels (>4.8–5.9%) [37]. Moreover, a 1% HbA_1c_ increase corresponds to an increase of 35mg/dL of average glucose [38]. In this variable, HIIT intervention was more effective than other interventions. It should be pointed out that 3 [22,26,28] of the 4 studies that compare HIIT to MIT, use a high weekly frequency and time of application, so that these two factors seem to be decisive for the achievement of the desired effects.

In addition to the positive effects produced by HIIT, compared to all other interventions, on the different variables except for diastolic BP, HIIT could have advantages compared to other types of training in terms of the short time in which it can be carried out. This is a very important aspect due to the limited time for exercise and leisure that we have in the society in which we are living. Moreover, the use of monitoring devices is recommended, making it possible to monitor the patients remotely so that the exercise could be carried out under the recommendations given by the professionals [39]. This could have an impact on the improvement of the patients’ adherence to the treatment, as demonstrated by various authors [20,22]. Finally, we emphasize that the outcome obtained in this study could be useful for the creation of future clinical practice guidelines that incorporate HIIT as physical exercise, in addition to advice on diet and healthy lifestyle habits.

## 5. Conclusions

This meta-analysis presents a current view on the effectiveness of HIIT in patients with T2D. The results obtained suggest that HIIT intervention, compared to MIT, LIT and CON, turns out to be effective in the improvement of the anthropometric conditions (body weight, BMI), cardiovascular conditions (systolic BP and VO2max) and metabolic conditions (HbA_1c_) in subjects with T2D. In addition, the results suggest that MIT intervention could be more effective than LIT and CON. We cannot draw any firm conclusions about the effectiveness of HIIT on diastolic BP.

The findings of this study recommend the incorporation of physical exercise through HIIT in the treatment of subjects with T2D. However, it is necessary to promote new lines of research in order to identify the most effective protocols according to their frequency, session duration and rehabilitation program duration, as well as a detailed description of the exercises.

## 6. Limitations

Despite having carefully selected the keywords and search strategies, there is the possibility that scientific literature of potential utility has been excluded from this review. Other possible limitations are the sample sizes used in the studies, the limited number of RCTs found and included in the meta-analysis that affects the meta-analysis groups composed of a few studies. The statistical results of the meta-analysis performed show heterogeneity for some outcomes. We hypothesize that it could be due to the heterogeneity in the interventions protocols conducted in the studies included with differences in the exercise modalities and methods used to determine the intensity desired, number and duration of intervals, session frequency and duration, use of active or passive recovery, and total intervention duration. Moreover, there is a lack of clear information on some data around the sample, for example, the physical activity level of the participants, gender, age, nutritional status, etc. Therefore, the results obtained from this statistical analysis must be treated with caution. In order to provide evidence for clinical practice, the results shown in this meta-analysis demonstrate a need for more research with greater methodological rigor using larger sample sizes, and to determine which exercise modality most positively affects anthropometric, cardiopulmonary and metabolic markers in individuals with T2D.

## Figures and Tables

**Table 1:** Physiotherapy Evidence Database (PEDro) scale score for clinical trials included in the review.

PEDro Scale												
Study	Total Score	1	2	3	4	5	6	7	8	9	10	11
Karstoff et al., 2013 [20]	6	-	1	0	1	0	0	0	1	1	1	1
Álvarez et al., 2016 [21]	6	-	1	0	1	0	0	1	1	0	1	1
Terada et al., 2012 [22]	7	-	1	0	1	0	0	1	1	1	1	1
Mitranum et al., 2012 [23]	5	-	1	0	1	0	0	0	1	0	1	1
Cassidy et al., 2015 [24]	5	-	1	0	1	0	0	0	1	0	1	1
Ruffino et al., 2016 [25]	5	-	1	0	1	0	0	0	1	0	1	1
Støa et al., 2016 [26]	5	-	0	1	1	0	0	0	1	0	1	1
Maillard et al., 2016 [27]	5	-	1	0	1	0	0	0	1	0	1	1
Hollekim-Strand et al., 2014 [28]	5	-	1	0	1	0	0	0	0	1	1	1
Balducci et al., 2012 [29]	5	-	1	0	1	0	0	0	1	0	1	1

**Table 2:** Main characteristics of participants in the studies.

Study	Groups	No. of Males/ Females	Average Age (Years) Mean (SD)	Years after Diagnosis Mean (SD)	Average Weight (Kg) Mean (SD)	Average Height (cm) Mean (SD)	Comorbidity among the Participants
Karstoff et al., 2013 [20]	HIIT (*n* = 12)	7/5	57.5 (2.4)	3.5 (0.7)	84.9 (4.9)	NA	None
	LIT (*n* = 12)	8/4	60.8 (2.2)	6.2 (1.5)	88.2 (4.7)		
	CON (*n* = 8)	5/3	57.1 (3)	4.5 (1.5)	88.5 (4.7)		
Álvarez et al., 2016 [21]	HIIT (*n* = 13)	0/13	45.6 (3.1)	3.4 (1.1)	73.8 (2)	156 (2)	None
	CON (*n* = 10)	0/10	43.1 (1.5)	3.6 (1.1)	75.3 (1.6)	158 (2)	
Terada et al., 2012 [22]	HIIT (*n* = 7)	4/4	62 (3)	6 (4)	80.5 (9.9)	NA	None
	MIT (*n* = 8)	4/3	63 (5)	8 (4)	93.9 (18.3)		
Mitranum et al., 2013 [23]	HIIT (*n* = 14)	5/9	61.2 (2.8)	19.5 (0.4)	66.5 (3.7)	149 (4)	None
	LIT (*n* = 14)	5/9	61.7 (2.7)	20.5 (0.4)	65.8 (3.1)	149 (5)	
	CON (*n* = 15)	5/10	60.9 (2.4)	21.1 (0.6)	67.7 (3.2)	152 (5)	
Cassidy et al., 2015 [24]	HIIT (*n* = 12)	10/2	61 (9)	5 (3)	90 (15)	171 (8)	None
	CON (*n* = 11)	8/3	59 (9)	4 (2)	90 (9)	169 (9)	
Ruffino et al., 2016 [25]	HIIT (*n* = 16)	16/0	55 (5)	4 (4)	96.7 (11.7)	178 (6)	None
	MIT (*n* = 16)				97 (11.6)	178 (6)	
Støa et al., 2016 [26]	HIIT (*n* = 19)	15/23	59 (11)	9 (7)	95 (15.3)	172 (6)	None
	MIT (*n* = 19)		59 (10)	6 (5)	89.1 (15.6)	170 (6)	
Maillard et al., 2016 [27]	HIIT (*n* = 8)	0/8	68.2 (1.9)	14.5 (2.1)	79.5 (5.2)	NA	None
	MIT (*n* = 9)	0/9	70.1 (2.4)		73.9 (3.4)		
Hollekim-Strand et al., 2014 [28]	HIIT (*n* = 20)	12/8	58.6 (5)	4.2 (2.3)	NA	NA	All the patients presented diastolic dysfunction of left ventricle.
	MIT (*n* = 17)	11/6	54.7 (5.3)	3 (2.6)			
Balducci et al., 2012 [29]	HIIT (*n* = 152)	91/61	59.5 (8.3)	7.8 (6.2)	NA	NA	None
	LIT (*n* = 136)	83/53	58.4 (8.9)	5.9 (4)			

HIIT—high-intensity interval training, LIT—low-intensity training, CON—control group, SD—standard deviation, NA—not available.

**Table 3:** Main characteristics of the study interventions.

Study	Intervention	Frequency	Session Duration	Intervention Duration	Outcome Measure	Measuring Instrument	Results
Karstoff et al., 2013 [20]	G1 (HIIT): Interval walking training with 3-min repetitions at low (<70% peak energy-expenditure rate) and high (>70%) intensity. G2 (LIT): continuous-walking training (<55%) G3 (CON): Non-Intervention	5 times/week	60 min	16 weeks	HbA_1c_ (%); Weight and BMI; VO_2max._; Systolic and Diastolic BP.	Blood sample through HPLC; DXA Scanner; Stress test.	Statistical differences were found in the LIT group: VO2max_._ (*p* < 0.001), Weight and BMI (*p* < 0.001).
Álvarez et al. 2016 [21]	G1 (HIIT): running/jogging (90–100% HRmax). 8–14 repetitions, active rest between sets (<70% HRmax) G2 (CON): Non-Intervention	3 times/week	22–37.5 min	16 weeks	HbA_1c_ (%); Systolic and diastolic BP; Weight; BMI.	Blood sample through Variant II of HPLC; OMROM *BP* automatic monitor; OMROM digital precision balance; P/H^2^.	Statistical differences were found in the HIIT group: Weight (*p* < 0.05), BMI (*p* < 0.05), Systolic BP (*p* < 0.05), and HbA_1c_ (*p* < 0.001).
Terada et al., 2012 [22]	G1 (HIIT): treadmill training or cycling intervals 1′ (100%VO2max)_._ and 3′ (20%VO2max). G2 (MIT): continuous treadmill training or cycling (40% VO2max).	5 times/week	30–60 min	12 weeks	Weight; BMI; VO2max_._; % Body fat; HbA_1c_ (%).	Stress test through treadmill and metabolic measurement system (True Max); P/H^2^; DXA Scanner; Blood sample.	Statistical differences were found in % Body fat (*p* = 0.009).
Mitranum et al., 2013 [23]	G1 (HIIT): 4–6 intervals (85% VO2max) during 1 min following 4 min of active rest (50% VO2max_._). G2 (LIT): 50–65% VO_2máx_. G3 (CON): Non-Intervention	3 times/week	30–40 min	12 weeks	Weight, BMI and % Body fat; VO2max_._; HR; Systolic and diastolic BP.	Bioelectrical impedance; Stress test (Modified Bruce protocol); PolarTeam 2 Pro monitor; BP monitor.	Statistical differences (*p* < 0.05) were found in Weight, BMI, % Body fat, Systolic BP, Heart rate and VO2max.
Cassidy et al., 2015 [24]	G1 (HIIT): 3 × 3′ cycloergometry G2 (CON): Non-intervention.	3 times/week	21–31 min	12 weeks	HbA_1c_ (%); Weight; Systolic and diastolic BP; Heart rate.	TOSOH HLC-723G8 analyzer; Plethysmography; Vascular unloading technique.	Nonstatistical differences were found.
Ruffino et al. 2016 [25]	G1 (HIIT): cycloergometry (86–88% HRmax). 2 sprints of 10–20′’. G2 (MIT): Walking (40–55% HRmax).	3 times/week 5 times/week	10 min 30 min	8 weeks	VO2max_._; Weight and % Body fat; Systolic and diastolic BP.	TrueOne 2400 gas analysis system; DXA Scanner; Alvita MC101 Monitor.	Statistical differences (*p* < 0.05) were found in Systolic and diastolic BP.
Støa et al., 2016 [26]	G1 (HIIT): 4 × 4′ (85–95% HRmax) with 3′ active rest (70% HRmax). G2 (MIT): 70–75% HRmax.	3 times/week	52 min 60 min	12 weeks	Weight; % Body fat; BMI; Systolic and diastolic BP; VO2max_._; HbA_1c_ (%).	Tefal Sensitive Computer; skin firmly; P/H^2^; Stethoscope and BP measurement; Stress test; Polar rs100.	Statistical differences were found in Weight (*p* < 0.01), % Body fat (*p* < 0.001), BMI (*p* < 0.001), HbA_1c_ (*p* < 0.001), VO2max_._ (*p* < 0.001), Diastolic BP (*p* < 0.01).
Maillard et al., 2016 [27]	G1 (HIIT): cycloergometry (77–85% HRmax). G2 (MIT): cycloergometry (55–60% HRmax).	2 times/week	30 min 50 min	16 weeks	Weight; BMI; % Body fat; HbA_1c_ (%).	sRCT 709 weighing scale; P/H^2^; DXA Scanner; Variant II Analyzer of HPLC.	Nonstatistical differences were found.
Hollekim-Strand et al., 2014 [28]	G1 (HIIT): 4 × 4′ (90–95% HRmax). G2: MIT	3 times/week 210 min./week	40 min ≥10 min	12 weeks	VO2max_._; HR; Systolic and diastolic BP; HbA_1c_ (%); BMI; % Body fat.	Not showed in study.	Statistical differences were found in VO2max (*p* < 0.001).
Balducci et al., 2012 [29]	G1 (HIIT): aerobic training (70% VO2max) + resistance training (60% 1-Repetition Maximum). G2 (LIT): aerobic training (55% VO2max_._) + resistance training (60% 1-Repetition Maximum).	2 times/week	64–70 min 76–83 min	48 weeks	HbA_1c_ (%); VO2max_._; BMI; Systolic and diastolic BP.	Blood biochemical test; Stress test through FitMate.	Statistical differences (*p* < 0.001) were found in: VO2max_._, BMI, Systolic and diastolic BP, HbA_1c_.

G—group, HIIT—high-intensity interval training, MIT—moderate-intensity training, LIT—low-intensity training, CON—control, VO2max—maximum oxygen uptake; HbA_1c_ (%)—hemoglobin A 1c, BMI—body mass index, BP—blood pressure, HPLC—high-performance liquid chromatography, DXA—dual-energy X-ray absorptiometry, HR—heart rate.

## Supplementary Materials

The following are available online at https://www.mdpi.com/1660-4601/16/22/4524/s1, Table S1: Search strategy, Table S2: Classification of the studies according to the type of interventions, comparison groups and outcomes.
