# Supplementary material for: Anthropometric, Cardiopulmonary and Metabolic Benefits of the High-Intensity Interval Training Versus Moderate, Low-Intensity or Control for Type 2 Diabetes: Systematic Review and Meta-Analysis
Source: Int J Environ Res Public Health. 2019 Nov 15;16(22):4524. doi: 10.3390/ijerph16224524 (PMC6887993; doi:10.3390/ijerph16224524)
Supplement: Supplementary file 1 [file ijerph-16-04524-s001.pdf]

Table S1. Search strategy.

Databases	Total articles	Search
PubMed	55	(High-intensity interval training OR High-intensity intermittent) AND Diabetes mellitus type 2
	10	("High-Intensity Interval Training"[Mesh]) AND "Diabetes Mellitus, Type 2"[Mesh]
PEDro	5	High-intensity interval training AND Diabetes mellitus type 2
WoS	129	TS=((“High-Intensity Interval Training*” OR “Interval Training, High-Intensity” OR “Interval Training, High-Intensity*” OR “Training, High-Intensity Interval” OR “Training, High-Intensity Interval*” OR “High-Intensity Intermittent Exercise” OR “Exercise, High-Intensity Intermittent” OR “Exercise, High-Intensity Intermittent*” OR “High-Intensity Intermittent Exercise*” OR “Sprint Interval Training” OR “Sprint Interval Training* OR “high-intensity interval”) AND (“Diabetes Mellitus, Noninsulin-Dependent” OR “Diabetes Mellitus, Ketosis-Resistant” OR “Diabetes Mellitus, Ketosis Resistant” OR “Ketosis-Resistant Diabetes Mellitus” OR “Diabetes Mellitus, Non-Insulin Dependent” OR “Diabetes Mellitus, Non-Insulin-Dependent” OR “Non-Insulin-Dependent Diabetes Mellitus” OR “Diabetes Mellitus, Stable” OR “Stable Diabetes Mellitus” OR “Diabetes Mellitus, Type II” OR “NIDDM” OR “Diabetes Mellitus, Noninsulin Dependent” OR “Diabetes Mellitus, Maturity-Onset” OR “Diabetes Mellitus, Maturity Onset” OR “Maturity-Onset Diabetes Mellitus” OR “Maturity Onset Diabetes Mellitus” OR “MODY” OR “Diabetes Mellitus, Slow-Onset” OR “Diabetes Mellitus, Slow Onset” OR “Slow-Onset Diabetes Mellitus” OR “Type 2 Diabetes Mellitus” OR “Noninsulin-Dependent Diabetes Mellitus” OR “Noninsulin Dependent Diabetes Mellitus” OR “Maturity-Onset Diabetes” OR “Diabetes, Maturity-Onset” OR “Maturity Onset Diabetes” OR “Type 2 Diabetes” OR “Diabetes, Type 2” OR “Diabetes Mellitus, Adult-Onset” OR “Adult-Onset Diabetes Mellitus” OR “Diabetes Mellitus, Adult Onset”)).

Table S2. Classification of the studies according to the type of interventions, comparison groups and outcomes.

Group	Subgroups	Studies
HIIT vs CON	Body weight (Kg)	Karstoff et al., 2013 [20], Mitranum et al., 2014 [23], Álvarez et al., 2016 [21], Cassidy et al., 2016 [24]
	BMI	Karstoff et al., 2013 [20], Mitranum et al., 2014 [23], Álvarez et al., 2016 [21]
	Systolic BP	Karstoff et al., 2013 [20], Mitranum et al., 2014 [23], Álvarez et al., 2016 [21], Cassidy et al., 2016 [24]
	Diastolic BP	Karstoff et al., 2013 [20], Mitranum et al., 2014 [23], Álvarez et al., 2016 [21], Cassidy et al., 2016 [24]
	VO2max.	Karstoff et al., 2013 [20], Mitranum et al., 2014 [23]
	HbA_{1c} (%)	Karstoff et al., 2013 [20], Cassidy et al., 2016 [24]
HIIT vs MIT	Body weight (Kg)	Støa et al., 2017 [26], Maillard et al., 2016 [27], Ruffino et al., 2016 [25], Terada et al., 2013 [22]
	BMI	Terada et al., 2013 [22], Hollekim-Strand et al., 2014 [28], Maillard et al., 2016 [27], Støa et al., 2017 [26]
	Systolic BP	Hollekim-Strand et al., 2014 [28], Ruffino et al., 2016 [25], Støa et al., 2017 [26]
	Diastolic BP	Hollekim-Strand et al., 2014 [28], Ruffino et al., 2016 [25], Støa et al., 2017 [26]
	VO2max.	Terada et al., 2013 [22], Hollekim-Strand et al., 2014 [28], Støa et al., 2017 [26]
	HbA_{1c} (%)	Terada et al., 2013 [22], Hollekim-Strand et al., 2014 [28], Maillard et al., 2016 [27], Støa et al., 2017 [26]
HIIT vs LIT	Body weight (Kg)	Mitranum et al., 2014 [23], Karstoff et al., 2013 [20]
	BMI	Balducci et al., 2012 [29], Karstoff et al., 2013 [20], Mitranum et al., 2014 [23]
	Systolic BP	Balducci et al., 2012 [29], Karstoff et al., 2013 [20], Mitranum et al., 2014 [23]
	Diastolic BP	Balducci et al., 2012 [29], Karstoff et al., 2013 [20], Mitranum et al., 2014 [23]
	VO2max.	Balducci et al., 2012 [29], Karstoff et al., 2013 [20], Mitranum et al., 2014 [23]
	HbA_{1c} (%)	Balducci et al., 2012 [29], Karstoff et al., 2013 [20]
